# Prevalence and factors associated with depressive, anxiety and stress symptoms among women with gestational diabetes mellitus in tertiary care centres in Malaysia: a cross-sectional study

**DOI:** 10.1186/s12884-019-2519-9

**Published:** 2019-10-21

**Authors:** Kai Wei Lee, Siew Mooi Ching, Fan Kee Hoo, Vasudevan Ramachandran, Seng Choi Chong, Maiza Tusimin, Noraihan Mohd Nordin

**Affiliations:** 1 Department of Family Medicine, Faculty of Medicine and Health Sciences, Universiti Putra Malaysia, Serdang, Malaysia; 2 Malaysian Research Institute on Ageing, Faculty of Medicine and Health Sciences, Universiti Putra Malaysia, Serdang, Malaysia; 3 Department of Medical Sciences, School of Healthcare and Medical Sciences, Sunway University, Bandar Sunway, Malaysia; 4 Department of Psychiatry, Faculty of Medicine and Health Sciences, Universiti Putra Malaysia, 43400 Serdang, Selangor Malaysia; 5 Department of Obstetrics and Gynaecology, Faculty of Medicine and Health Sciences, Universiti Putra Malaysia, 43400 Serdang, Selangor Malaysia; 6 Department of Obstetrics and Gynaecology, Hospital Kuala Lumpur, 50586 Kuala Lumpur, Malaysia

**Keywords:** Depression, Anxiety, Stress, Gestational diabetes, Malaysia

## Abstract

**Background:**

Research on antenatal depressive, anxiety and stress symptoms among women with gestational diabetes mellitus (GDM) is lacking in Malaysia. This study aimed to determine the prevalence and factors associated with antenatal depressive, anxiety and stress symptoms among Malaysian women with GDM.

**Methods:**

This was a descriptive, cross-sectional study of 526 women with GDM. Depressive, anxiety and stress symptoms are defined as the final score in mild to extremely severe risk in the severity rating scale. Data analysis was performed using SPSS v.21, while multiple logistic regression was used to identify predictors of depressive, anxiety and stress symptoms.

**Results:**

Prevalence of anxiety symptoms was highest (39.9%), followed by depressive symptoms (12.5%) and stress symptoms (10.6%) among women with GDM. According to multiple logistic regression analyses, younger age (OR = 0.955, 95% CI = 0.919–0.993), comorbidity with asthma (OR = 2.436, 95% CI = 1.219–4.870) and a family history of depression and anxiety (OR = 4.782, 95% CI = 1.281–17.853) had significant associations with antenatal anxiety symptoms. Being non-Muslim (OR = 2.937, 95% CI = 1.434–6.018) and having a family history of depression and anxiety (OR = 4.706, 95% CI = 1.362–16.254) had significant associations with antenatal depressive symptoms. Furthermore, being non-Muslim (OR = 2.451, 95% CI = 1.273–4.718) had a significant association with antenatal stress symptoms.

**Conclusions:**

Within a population of women with GDM in Malaysia, those at higher risk of having depressive, anxiety and stress symptoms can be identified from several baseline clinical characteristics. Clinicians should be more alert so that the high-risk patients can be referred earlier for further intervention.

## Introduction

Gestational diabetes mellitus (GDM) is one of the most common medical conditions encountered by pregnant women in Asia [1]. A diagnosis of GDM increases vulnerability to emotional distress, such as depression, anxiety or stress among pregnant women [2–5], as well as having an adverse impact on their self-perception towards health [3] and quality of life [6]. On the other hand, there are studies showing that a diagnosis of GDM is associated with improved scores of maternal wellbeing postpartum, as the treatment of GDM reduces serious morbidity [7]. The transition to motherhood is already a major life-changing event and also a stressful condition; when this transition is associated with diabetes, it will further exaggerate psychological stress on women [4, 8]. This is due to the disease of GDM always requiring patients’ self-determination to overcome the underlying shock of diagnosis and to strictly adhere to lifestyle modifications to prevent further disease complications [9]. Moreover, women with GDM are aware of the fact that uncontrolled diabetes can lead to pregnancy-related complications and poor neonatal outcome [7, 10]. Therefore, they suffer from a higher propensity for the development of antenatal depression, anxiety and stress [8, 11].

Studies have shown that the prevalence of depressive symptoms among women with GDM ranged from 25.9 to 56.7% [12, 13]; for anxiety, from 4.8 to 57.7% [14, 15]; and stress, from 19.3 to 62.8% [14, 16]. Depressive and anxiety symptoms in pregnancy have been associated with younger age groups, low education levels, unemployment, Asian ethnicities, low socioeconomic status, poor social support, heavy alcohol consumption and smoking [17–23]. Other risk factors include major life events, low self-esteem, and a previous history of depression [24]. Furthermore, pregnant women with a negative cognitive style, such as pessimism, anger and rumination are more likely to be associated with depression and anxiety. Other studies have also reported that pregnant mothers with an unplanned pregnancy, previous complications of pregnancy and a negative experience with previous pregnancies [23, 25–28] have an association with depression and anxiety. However, the results were conflicting for parity and gravidity.

Although information on prevalence and risk factors for antenatal depressive, anxiety and stress symptoms are abundant in the literature, there has been no study done in Asia, and particularly, in Malaysia, which is a multi-ethnic country. Undiagnosed and untreated antenatal depressive and anxiety symptoms among women with GDM could lead to postpartum depression and anxiety [29]. Therefore, this study aimed to determine the prevalence and factors associated with depressive, anxiety and stress symptoms among women with GDM in tertiary care centres in Malaysia.

## Methods

### Study design and setting

This was a cross-sectional study conducted in two public tertiary care centres, including the Hospital Kuala Lumpur and the Hospital Serdang. Hospital Kuala Lumpur is a referral healthcare centre in Malaysia, located in the centre of Kuala Lumpur. Hospital Serdang is a tertiary hospital that serves around 570,000 people in the Serdang, Putrajaya, Kajang and Bangi districts.

### Study population

Pregnant Malaysian women aged ≥18 years old, currently in the second or third trimester with underlying GDM, were recruited for this study. The diagnosis of GDM is defined as fasting plasma glucose ≥5.1 mmol/L or 75-g two-hour OGTT ≥7.8 mmol/L according to Malaysian Clinical Practice Guidelines [30, 31]. Patients with pre-existing diabetes were excluded from this study.

### Sample size calculation

The sample size was calculated using the Lemeshow et al. (1999) formula [32] based on the prevalence of depressive symptoms among women with GDM, which ranged from 24 to 42% [33]. The estimated sample size was 367 with 99% power, a 95% confidence interval (CI), and the statistical significance level (α) at 5%. The total number of respondents needed was 524 after considering a non-response rate of 30%.

### Sampling method

We recruited the study respondents using a systematic random sampling method. The estimated number of patients with GDM that visited the clinic per day totalled 20 patients. Furthermore, there were 5 days of antenatal clinics per week and over the course of 3 months, 1200 patients were seen. Since the number of required test subjects was 524, a sampling interval of two was used as the constant difference between subjects. The first starting number of two was picked randomly using the lottery method from the registration counter. We used a structured self-administered questionnaire to obtain socio-demographic and relevant clinical information from the respondents.

### Instruments

We used an English [34] and Malay [35] version of the validated questionnaire on Depression, Anxiety and Stress 21 items (DASS-21) in this study. DASS-21 [34] is the short version of Depression, Anxiety and Stress 42 items (DASS-42) [36]. The English version of the questionnaire (DASS-42) has a good validation with Cronbach’s alpha values of 0.91 for depression; 0.84 for anxiety; and 0.90 for stress [36], while DASS-21’s internal consistency for depression subscale is 0.72; anxiety subscale 0.77; stress subscale 0.70. The overall Cronbach’s alpha for DASS-21 is 0.88 [34]. The translated Malay version of the DASS-21 questionnaire has good Cronbach’s alpha values, as well as among the Malaysian population (0.84 for depression; 0.74 for anxiety; and 0.79 for stress) [35] and among diabetic patients (0.75 for depression; 0.74 for anxiety; and 0.79 for stress) [37].

The respondents were rated on a 4-point severity scale based on their experiences over the past week. The 4-points severity scale ranged from 0 (doesn’t apply to me); 1 (applied to me to some degree, or some of the time), 2 (applied to me to a considerable degree or a good part of the time) and 3 (applies to me most of the time). Scores for subscale for depression, anxiety and stress were calculated by summing the scores for the relevant items in DASS-21, and the values were multiplied by two to calculate the final score. Table 1 shows the cut-off values for severity rating scales of depression, anxiety, and stress. The depressive symptoms defined follow the depression subscale ≥10; anxiety symptoms, ≥8; and stress symptoms, ≥15 [36].

**Table 1:** Cut-off score for severity ratings of DASS-42 [36]

	Depression	Anxiety	Stress
Normal	0–9	0–7	0–14
Mild	10–13	8–9	15–18
Moderate	14–20	10–14	19–25
Severe	21–27	15–19	26–33
Extremely severe	28+	20+	34+

*Mild-Moderate-Severe-Extremely Severe were categorised as “With Symptoms” in regression analysis

### Statistical analysis

We used IBM SPSS Statistics version 21.0 to perform the data analysis. Categorical data were reported in terms of percentage and continuous data was described in mean. The association between categorical data was analysed with Chi-square test. The association between two sets of continuous data was analysed using an independent t-test. Multiple logistic regression analysis was used to identify the predictors of the depressive, anxiety and stress symptoms. Variables with a *p*-value of less than 0.25 in univariate analysis were entered into multiple logistic regression [38], for the fact that p-value set at < 0.05 may miss any variables known to be important [39, 40]. A backward stepwise regression method was used [41]. All analyses were made with a 95% CI, and the level of significance was set at *p* < 0.05.

## Results

### Respondents’ characteristics

A total of 526 respondents were recruited into this study. The mean age of respondents was 32.3 ± 4.9 years old (median was 32.0 ± 7). The majority were Malay (82.3%) and married (96.0%), while 7.8% had underlying asthma and 2.9% had a family history of depression and anxiety.

There were 66 (12.5%) women with depressive symptoms (5.1% mild, 5.5% moderate, 1.5% severe and 0.4% extremely severe); 210 (39.9%) women with anxiety symptoms (12% mild, 17.3% moderate, 5.7% severe and 4.9% extremely severe) and 56 (10.6%) women with stress symptoms (6.5% mild, 2.3% moderate, 1.5% severe and 0.3% extremely severe).

### Variables associated with depressive, anxiety and stress symptoms using univariate analysis

Table 2 shows the association between depressive, anxiety and stress symptoms and the clinical variables of the respondents using univariate analysis. For depressive symptoms, being non-Malay (*p* < 0.001), non-Muslim (p < 0.001), and having a history of allergies (*p* = 0.011) or a family history of depression and anxiety (*p* = 0.005) were found to be significantly associated with depressive symptoms among women with GDM.

**Table 2:** Association between symptoms of depression, anxiety and stress and clinical variables among women with GDM using univariate analysis (*n* = 526)

Parameters		Depressive Symptoms			Anxiety Symptoms			Stress Symptoms		
		No *n* = 460 (87.5%)	Yes *n* = 66 (12.5%)	*p*-value	No *n* = 316 (60.1%)	Yes *n* = 210 (39.9%)	*p*-value	No *n* = 470 (89.4%)	Yes *n* = 56 (10.6%)	*p*-value
A Diagnosis of Gestational Diabetes Mellitus and Treatment Profile										
Baseline Fasting Plasma Glucose, mmol/L		5.48 ± 1.43	5.52 ± 1.40	0.877	5.50 ± 1.46	5.48 ± 1.38	0.897	5.48 ± 1.43	5.58 ± 1.38	0.619
Baseline 75-g Two-Hour OGTT, mmol/L		9.13 ± 2.52	9.18 ± 2.87	0.895	9.09 ± 2.50	9.22 ± 2.66	0.577	9.12 ± 2.51	9.32 ± 2.99	0.584
Baseline HbA1c at Diagnosis, %		5.87 ± 1.47	5.57 ± 0.93	0.311	5.87 ± 1.57	5.80 ± 1.18	0.672	5.85 ± 1.46	5.72 ± 0.95	0.679
Treatments	OAD and/or diet modification	319(87.6)	45(12.4)	0.809	225(61.8)	139(38.2)	0.231^a^	327(89.8)	37(10.2)	0.560
	Insulin with/out OAD and/or diet modification	139(86.9)	21(13.1)		90(56.3)	70(43.8)		141(88.1)	19(11.9)	
Self-Monitoring with Glucometer	No	61(82.4)	13(17.6)	0.074 ^a^	42(56.8)	32(43.2)	0.405	65(87.8)	9(12.2)	0.476
	Yes	323(89.7)	37(10.3)		223(61.9)	137(38.1)		326(90.6)	34(9.4)	
Socio-Demographic Factors										
Age		32.36 ± 4.93	31.73 ± 4.67	0.330	32.64 ± 4.92	31.73 ± 4.82	0.037	32.34 ± 4.88	31.73 ± 5.04	0.379
Ethnicity	Malay	391(90.3)	42(9.7)	0.000 ^a^	270(62.4)	163(37.6)	0.021^a^	395(91.2)	38(8.8)	0.003 ^a^
	Non-Malay	69(74.2)	24(25.8)		46(49.5)	47(50.5)		75(80.6)	18(19.4)	
BMI, kg/m^2^		29.51 ± 6.28	28.70 ± 5.83	0.331	29.37 ± 5.91	29.44 ± 6.65	0.899	29.38 ± 6.09	29.52 ± 7.26	0.880
Religion	Muslim	398(90.2)	43(9.8)	0.000 ^a^	274(62.1)	167(37.9)	0.037^a^	402(91.2)	39(8.8)	0.002 ^a^
	Non-Muslim	61(72.6)	23(27.4)		42(50.0)	42(50.0)		67(79.8)	17(20.2)	
Education	Secondary and below	205(85.1)	36(14.9)	0.134 ^a^	145(60.2)	96(39.8)	0.903	211(87.6)	30(12.4)	0.275
	Tertiary	246(89.5)	29(10.5)		164(59.6)	111(40.4)		249(90.5)	26(9.5)	
Employment	Unemployed	152(85.9)	25(14.1)	0.371	110(62.1)	67(37.9)	0.497	156(88.1)	21(11.9)	0.500
	Employed	303(88.6)	39(11.4)		202(59.1)	140(40.9)		308(90.1)	34(9.9)	
Family Income, Ringgit Malaysia		3946.02 ± 2402.72	3809.79 ± 3232.09	0.758	3887.51 ± 2437.18	3990.73 ± 2632.08	0.662	3916.01 ± 2405.89	4038.82 ± 3313.27	0.798
Pregnancy Planned	No	296(87.3)	43(12.7)	0.858	207(61.1)	132(38.9)	0.758	302(89.1)	37(10.9)	0.981
	Yes	144(86.7)	22(13.3)		99(59.6)	67(40.4)		148(89.2)	18(10.8)	
Marital Status	Without husband	16(76.2)	5(23.8)	0.162 ^b^	15(71.4)	6(28.6)	0.286	17(81.0)	4(19.0)	0.259
	With husband	441(88.2)	59(11.8)		299(59.8)	201(40.2)		450(90.0)	50(10.0)	
Parity	Nulliparous- Primiparous	246(87.5)	35(12.5)	0.973	163(58.0)	118(42.0)	0.229	252(89.7)	29(10.3)	0.837
	Multiparous ≥2	209(87.4)	30(12.6)		151(63.2)	88(36.8)		213(89.1)	26(10.9)	
Smoking	No	443(87.0)	66(13.0)	1.000	301(59.1)	208(40.9)	0.086^b^	454(89.2)	55(10.8)	1.000
	Yes	6(100.0)	0(0.0)		6(100.0)	0(0.0)		6(100.0)	0(0.0)	
Alcohol	No	449(87.7)	63(12.3)	1.000	309(60.4)	203(39.6)	0.719	459(89.6)	53(10.4)	0.587
	Yes	7(87.5)	1(12.5)		4(50.0)	4(50.0)		7(87.5)	1(12.5)	
Past Obstetric History										
Abortion	No	349(88.6)	45(11.4)	0.158 ^a^	244(61.9)	150(38.1)	0.140^a^	353(89.6)	41(10.4)	0.717
	Yes	109(83.8)	21(16.2)		71(54.6)	59(45.4)		115(88.5)	15(11.5)	
Preterm Delivery	No	441(87.7)	62(12.3)	0.321	305(60.6)	198(39.4)	0.233^a^	449(89.3)	54(10.7)	1.000
	Yes	17(81.0)	4(19.0)		10(47.6)	11(52.4)		19(90.5)	2(9.5)	
Gestational Diabetes Mellitus	No	356(88.1)	48(11.9)	0.366	245(60.6)	159(39.4)	0.650	366(90.6)	38(9.4)	0.082 ^a^
	Yes	102(85.0)	18(15.0)		70(58.3)	50(41.7)		102(85.0)	18(15.0)	
Current Medical Problems										
Hypertension	No	434(87.1)	64(12.9)	0.760	298(59.8)	200(40.2)	0.573	445(89.4)	53(10.6)	0.751
	Yes	24(92.3)	2(7.7)		17(65.4)	9(34.6)		23(88.5)	3(11.5)	
Allergy	No	451(88.1)	61(11.9)	0.011^b^	310(60.5)	202(39.5)	0.236^b^	460(89.8)	52(10.2)	0.030^b^
	Yes	7(58.3)	5(41.7)		5(41.7)	7(58.3)		8(66.7)	4(33.3)	
Asthma	No	424(87.8)	59(12.2)	0.368	298(61.7)	185(38.3)	0.011^a^	435(90.1)	48(9.9)	0.066^b^
	Yes	34(82.9)	7(17.1)		17(41.5)	24(58.5)		33(80.5)	8(19.5)	
Family History										
Heart Disease	No	390(88.6)	50(11.4)	0.052 ^a^	268(60.9)	172(39.1)	0.395	394(89.5)	46(10.5)	0.693
	Yes	68(81.0)	16(19.0)		47(56.0)	37(44.0)		74(88.1)	10(11.9)	
Depression and Anxiety	No	447(88.9)	56(11.1)	0.005^b^	308(61.2)	195(38.8)	0.029^a^	453(90.1)	50(9.9)	0.191^b^
	Yes	9(60.0)	6(40.0)		5(33.3)	10(66.7)		12(80.0)	3(20.0)	
Gestational Diabetes Mellitus	No	301(89.6)	35(10.4)	0.085^a^	208(61.9)	128(38.1)	0.335	307(91.4)	29(8.6)	0.089 ^a^
	Yes	151(84.4)	28(15.6)		103(57.5)	76(42.5)		155(86.6)	24(13.4)	

Data are presented as either n (%) or mean ± SD

^a^Pearson Chi-Square at *p* < 0.25 entered multivariate logistic regression

^b^Fisher’s Exact Test at *p* < 0.25 entered multivariate logistic regression

Anxiety symptoms showed that younger women (*p* = 0.037), being non-Malay (*p* = 0.021), non-Muslim (p = 0.037), having underlying asthma (p = 0.011), as well as a family history of depression and anxiety (*p* = 0.029) were found to be significantly associated with antenatal anxiety.

For stress symptoms, women who are Non-Malay (*p* = 0.003), non-Muslim (*p* = 0.002) and have a history with allergies (*p* = 0.030) were found to be significantly associated with antenatal stress.

### Predictors for depressive, anxiety and stress symptoms using multivariate analysis

Table 3 shows the predictors of depressive, anxiety and stress symptoms among women with GDM using multiple logistic regression analysis. Those who were non-Muslim [odds ratio (OR) =2.937, 95% confidence interval (CI) = 1.434–6.018] and had a family history of depression and anxiety (OR = 4.706, 95% CI = 1.362–16.254) were at higher odds of having depressive symptoms during the antenatal period.

**Table 3:** Predictors of depressive, anxiety and stress symptoms among women with GDM using multiple logistic regression (n = 526)

Parameters		Depressive Symptoms		Anxiety Symptoms		Stress Symptoms	
		Adjusted Odds Ratio (95% CI)	*p*-value	Adjusted Odds Ratio (95% CI)	*p*-value	Adjusted Odds Ratio (95% CI)	*p*-value
A Diagnosis of Gestational Diabetes Mellitus and Treatment Profile							
Self-Monitoring with Glucometer	No	1.743 (0.813–3.737)	0.153				
	Yes	Reference	–				
Treatments	OAD and/or diet modification			Reference	–		
	Insulin with/out OAD and/or diet modification			1.204 (0.803–1.806)	0.369		
Socio-Demographic Factors							
Age				0.955 (0.919–0.993)	0.020		
Ethnicity	Malay	Reference	–	Reference	–	Reference	–
	Non-Malay	0.537 (0.030–9.660)	0.673	1.533 (0.944–2.492)	0.084	0.492 (0.030–8.214)	0.622
Religion	Muslim	Reference	–	Reference	–	Reference	–
	Non-Muslim	2.937 (1.434–6.018)	0.003	0.891 (0.234–3.394)	0.866	2.451 (1.273–4.718)	0.007
Education	Secondary and below	1.168 (0.607–2.248)	0.641				
	Tertiary	Reference	–				
Marital Status	Without husband	1.285 (0.252–6.551)	0.763				
	With husband	Reference	–				
Parity	Nulliparous- Primiparous			0.968 (0.621–1.509)	0.886		
	Multiparous ≥2			1.000	–		
Past Obstetric History							
Abortion	No	Reference	–	Reference	–		
	Yes	1.251 (0.585–2.675)	0.563	1.351 (0.878–2.078)	0.171		
Preterm Delivery	No			Reference	–		
	Yes			1.901 (0.760–4.753)	0.169		
Gestational diabetes mellitus						Reference	–
						1.351 (0.700–2.605)	0.370
Current Medical Problems							
Allergy	No	Reference		Reference	–	Reference	–
	Yes	2.543 (0.455–14.208)	0.288	1.634 (0.421–6.346)	0.478	3.224 (0.781–13.304)	0.106
Asthma	No			Reference	–	Reference	–
	Yes			2.436 (1.219–4.870)	0.012	1.966 (0.806–4.798)	0.137
Family History							
Heart Disease	No	Reference	–				
	Yes	2.015 (0.944–4.302)	0.070				
Depression and Anxiety	No	Reference	–	Reference	–	Reference	–
	Yes	4.706 (1.362–16.254)	0.014	4.782 (1.281–17.853)	0.020	1.949 (0.510–7.451)	0.329
Gestational Diabetes Mellitus	No	Reference	–			Reference	–
	Yes	1.258 (0.646–2.449)	0.500			1.521 (0.844–2.739)	0.163

*Significant at *p* < 0.05

Younger age was associated with antenatal anxiety (OR = 0.955, 95% CI = 0.919–0.993). Those with underlying asthma (OR = 2.436, 95% CI = 1.219–4.870) and having a family history of depression and anxiety (OR = 4.782, 95% CI = 1.281–17.853) were at risk of having antenatal anxiety.

Furthermore, being a non-Muslim (OR = 2.451, 95% CI = 1.273–4.718) was the only predictor of antenatal stress.

We subsequently examined the population according to whether they were Muslim or non-Muslim, as this was a significant factor associated with depressive and stress symptoms. We found that a higher percentage of Muslim women received tertiary education (55.3%) compared with non-Muslim women (43.4%) in our study (*p* = 0.046). There was also a higher percentage of non-Muslim women (8.3%) receiving insulin therapy with or without OAD compared with Muslim women (3.0%) in our study (*p* = 0.029). Additionally, a higher percentage of Non-Muslim women (65.1%) were nulliparous or primiparous compared with Muslim women (51.8%) in our study population (*p* = 0.027). Lastly, drinking alcohol (*p* = 0.025) with underlying allergies (*p* = 0.030) and with a family history of depression and anxiety (*p* = 0.018) was more common among non-Muslim women compared to Muslim women.

## Discussion

In this study, the prevalence of depressive symptoms was 12.5%, 39.9% for anxiety symptoms and 10.6% for stress symptoms in women with GDM. The predictors for depressive symptoms included being non-Muslim and those with a family history of depression and anxiety. Predictors for anxiety symptoms were being a younger age, those with a family history of depression and anxiety, and having asthma. Additionally, being a non-Muslim was the predictor for stress symptoms.

The prevalence of depressive symptoms in this study is lower than a study done in Brazil, where the prevalence was 31% [33]. That study was done in Brazil and conducted by Dame et al. among women with GDM in multiple tertiary hospital settings [33]. A possible reason for these differences could be due to a different screening tool used [33]. We used DASS-21 to screen depressive symptoms, while Dame et al. used the Edinburgh Postnatal Depression Scale for depressive symptoms.

The prevalence of anxiety symptoms in this study is lower than a study conducted by Egan et al., 2017, which was 57.7% [14]. This discrepancy could be attributed to the higher percentage of the study population with hypertension (12.7%) in Egan et al. in comparison to 5% of those with hypertension in this study. A prior study reported that there was higher anxiety among hypertensive patients than those without hypertension (OR = 1.18, 95% CI = 1.02–1.37) [42].

Furthermore, the prevalence of stress symptoms in this study is much lower than the study findings in Egan et al. 2017 (62.8%) among the population in Ireland [14] and 19.3% were found in a study by Ahmed et al., among the Saudi Arabian population [16]. A possible reason for the higher stress symptoms observed in Egan et al. might also due to a higher percentage of the study population with hypertension. A meta-analysis reported that hypertension increased the odds of psychosocial stress (OR = 2.69, 95% CI = 2.32–3.11) compared to the normotensive population [43]. Another possible explanation for this is that Ahmed et al. used the Perceived Stress Scale to evaluate psychosocial stress levels.

Our study has shown that non-Muslim pregnant women with GDM have higher odds of having depressive and stress symptoms compared to Muslims. Firstly, these results could be due to the fact that there was a higher percentage of Muslim women (55.3%) receiving tertiary education compared to non-Muslim women (43.4%). Previous literature has reported that those with a higher education level are more established in their career and are financially more secure; therefore, they are at lower risk of having any mental distress [18, 44, 45]. Secondly, there was a higher percentage of non-Muslims (39.3%) in this study receiving insulin with and without OAD compared to Muslims (29.0%). Literature reviews have shown there is a significant association between insulin and hypoglycaemia attacks [46, 47]. Therefore, diabetic patients on insulin or OAD are more prone to getting depression and stress. There is also a higher percentage of Non-Muslims (65.1%) that were nulliparous or primiparous compared with Muslims (51.8%) in our study population (*p* = 0.027). Previous studies found a significant correlation between antenatal depression and nulliparous or primiparous [48, 49]. Similarly, drinking alcohol (*p* = 0.025) with underlying allergies (*p* = 0.030) and a family history of depression and anxiety (*p* = 0.018) was more common among non-Muslim women compared to Muslim women. The study also showed that antenatal depression is more prevalent among those who drink alcohol [44] and have a chronic medical condition, such as allergies and a family history of mental illness [45, 50].

Our study shows that younger women with GDM were at higher risk of developing anxiety. This is consistent with other studies [18–20, 25, 51]. This could be due to the fact that those younger expectant mothers were inexperienced in coping with pregnancy-related symptoms and lifestyle adjustments in disease management.

A family history of depression and anxiety was found to be one of the salient factors for developing depressive and anxiety symptoms in our study, and this is consistent with previous studies [45, 50]. A possible reason is that those with a family history of depression and anxiety inherited the gene of the psychiatric disorder [52–55] and it could be manifested when they are in a stressful situation, especially after the diagnosis of GDM.

This study found that asthma is one of the significant predictors for antenatal anxiety symptoms among women with GDM. This finding is consistent with other studies that reported that those with asthma are at risk of getting anxiety [56–58]. Being a GDM patient, they are already burdened with the disease and need to have a strict control over their diet or close monitoring of their glucose levels. Their anxiety levels will be further exacerbated by underlying asthma. This highlights the importance of emotional distress screenings in a group of patients to ensure a good obstetrical outcome.

### Strength and limitations

To date, this is the first comprehensive study to determine depressive, anxiety and stress symptoms among women with GDM in Malaysia. This study aimed to specifically examine the prevalence and factors associated with antenatal depressive, anxiety and stress symptoms solely among women with GDM. Previous studies have reported the prevalence and factors associated with depression and anxiety in pregnancy only but not among women with GDM. Furthermore, our study was conducted in two tertiary centres in Malaysia and the sample size of our study was greater than 500 (*n* = 526).

This study has some limitations; firstly, DASS-21 is a screening tool and the diagnosis of depression, anxiety and stress need to be confirmed by a mental health clinician. Furthermore, we did not capture the onset of the diagnosis of GDM. Secondly, we did not measure symptoms of depression, anxiety and stress either before pregnancy, prior to the GDM diagnosis, or among pregnant women without a GDM diagnosis. Therefore, we can only report that there is an association but are not able to determine the causal relationship between GDM and the presence of depression, anxiety or stress symptoms.

The implication from this study is that the physicians who treat women with GDM comorbid with asthma and a family history of depression and anxiety should remain vigilant for the presence of emotional distresses, and carefully evaluate whether there is a clinical need to treat each condition.

## Conclusion

Nearly two-fifths of women with GDM had anxiety symptoms, followed up by one-tenth of them having depressive and stress symptoms. Within a population of women with GDM in Malaysia, those at higher risk of having depressive, anxiety and stress symptoms can be identified from several baseline clinical characteristics. Hence, in managing patients with GDM, the clinician should be more alert and identify patients with depressive, anxiety and stress symptoms so that they can be referred earlier for further interventions.

## Data Availability

The datasets used and/or analysed during the current study are available from the corresponding author on reasonable request.

## Abbreviations

CI: Confidence interval
DASS-21: Depression, anxiety and stress scale 21-items
DASS-42: Depression, anxiety and stress scale 42-items
GDM: Gestational diabetes mellitus
OAD: Oral anti-diabetic drugs
OGTT: Oral glucose tolerance test
OR: Odds ratio
